# Posterior tibial nerve schwannoma in a multiple myeloma patient: A case report

**DOI:** 10.1177/2050313X19838441

**Published:** 2019-04-10

**Authors:** Michael Daniel, David Waters, Chaoyang Chen, Nicole Brouyette

**Affiliations:** 1 Podiatric Surgery, Ascension St. John Hospital, Detroit, MI, USA; 2 Division of Foot and Ankle, Henry Ford Health System, Detroit, MI, USA; 3 Department of Orthopedic Surgery, Henry Ford Hospital, Detroit, MI, USA

**Keywords:** Surgery, neurology, orthopedics/rehabilitation/occupational therapy

## Abstract

We report the case of a 61-year-old man with sharp pain, tender mass at the left lower one-third posterior tibial region. The patient has a 10-year history of multiple myeloma and related chemotherapy. A positron emission tomography computed tomography and magnetic resonance imaging indicated the suspect of a posterior tibial nerve tumor. En bloc resection of the tumor was performed under guidance of nerve stimulator without resecting nerve trunk. Active nerve was reserved without any neuropathic pain. Histology revealed the presence of a peripheral schwannoma. In conclusion, the tibial nerve schwannoma appeared to be a whole nerve mass, but meticulous dissection showed that this tibial nerve schwannoma was a well-encapsulated tumor and can be separated from the nerve trunk with active nerve fibers reserved. Removal of the tumor made patient free of pain and asymptomatic after surgery. The level of clinical evidence is 4.

## Introduction

Schwannomas are benign nerve sheath tumors that can be associated with genetic syndromes such as neurofibromatosis type 2 (NF2) or can occur as a result of therapeutic irradiation. The location of the schwannoma and the associated nerve can impact the quality of life. Schwannomas can be associated with pain, paresthesia, and motor weakness.^1^ The exact mechanism of schwannoma development is not fully understood; one theory suggests that axons of peripheral nerves exposed to repetitive mechanical trauma from nerve compression and irritation are not able to realign properly or contact with the injured axons, leading to Schwann cells remaining in a continuous proliferative state.^2^ Schwannomas can also be caused by loss of the NF2 tumor suppressor gene leading to the development of multiple tumors of the nervous system including schwannomas.^3^ Schwannomas occur most commonly in the head and neck region, and are rarely found in the extremities, but when they do occur in the extremities, they tend to affect the sciatic or ulnar nerves.^4^ There are only a few cases reported in the literature on tibial nerve schwannomas caused by injury.^4^ There is not a case report regarding the presentation of a posterior tibial nerve schwannoma in patients with advanced malignant processes of multiple myeloma.

Herein, we present an unusual case of a posterior tibial nerve schwannoma in a patient with a history of 10 years of multiple myeloma and chemotherapy, which was diagnosed in a close cooperation of the radiologist, orthopedic surgeon, and pathologist.

## Case report

Our patient is a 61-year-old Caucasian male, with a 10-year history of multiple myeloma (IgM kappa multiple myeloma). He presented to clinic with a sharp pain at left lower one-third tibial region with a small mass (15 × 15 × 10 mm^3^) palpated, he rated the pain score up to 10/10 based on visual analog scale and described it as sharp shooting pain that radiates to his toes and occasionally goes up to the proximal leg. On physical exam, there is a palpable nodule proximal to the medial malleolus with a positive Tinel’s sign upon percussion of the tibial nerve. Pain has been going on for approximately 7 years and has been progressively worsening over time.

The patient was treated by chemotherapy using fludarabine and rituximab in 2009 without response, then followed by a combination of bortezomib and dexamethasone, which brought in an excellent partial remission. Currently, he was treated by trametinib and lenalidomide.

**Figure 1.**
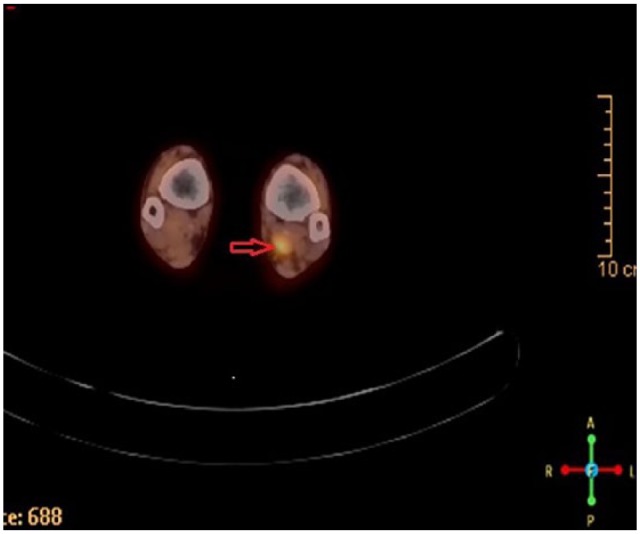
PET/CT shows hypermetabolic focus in the soft tissues of the posterior left medial ankle (arrow).

**Figure 2.**
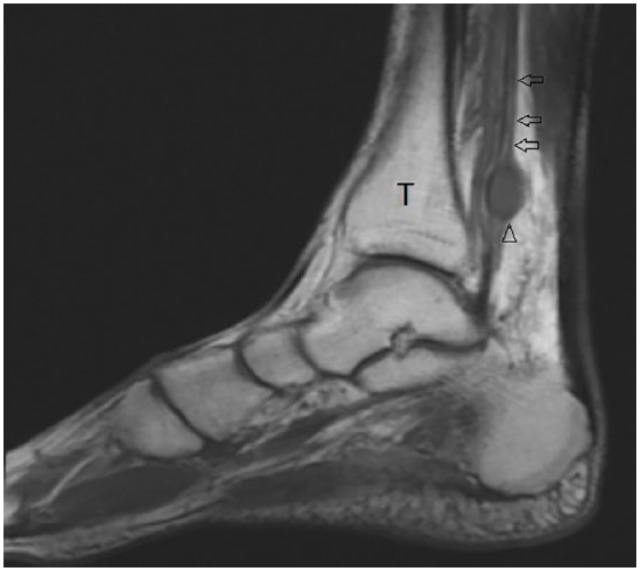
Sagittal T1 MRI image, showing tibial nerve lesion (arrow head) and posterior tibial nerve (arrows) and tibia bone (T).

**Figure 3.**
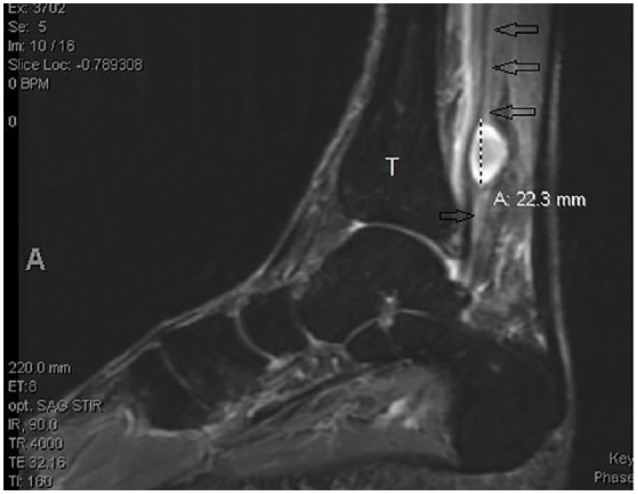
Sagittal STIR (short-TI inversion recovery) image showing tibial nerve lesion with a longitudinal diameter of 22.3 mm, posterior tibial nerve (arrows), and tibia bone (T).

**Figure 4.**
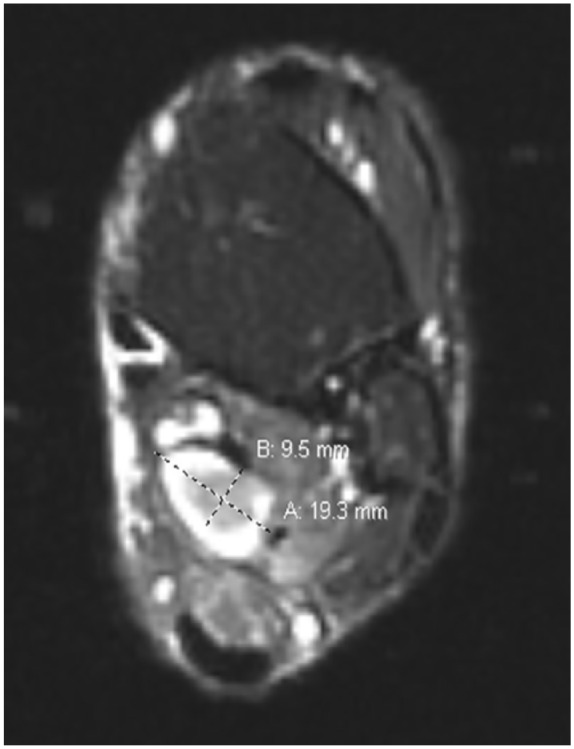
Axial T2 MRI image showing a well-defined hyperintense tibial nerve lesion measuring 9.5 mm × 19.3 mm.

Approximately 4 months before presenting to clinic, the patient was examined by a whole-body positron emission tomography computed tomography (PET/CT) scanning to monitor the metastatic progression of multiple myeloma and response to chemotherapy. PET/CT showed a hypermetabolic focus in the soft tissues of the posterior left medial ankle (Figure 1). The lesion went undiagnosed by the oncologist and radiologist and the patient continued to experience pain in the region until a magnetic resonance imaging (MRI) scanning was performed 2 months after the PET/CT was obtained. The left ankle contrast MRI showed a T1 hypointense (Figure 2) with mixed T2 hyperintense (Figure 3) mass lesion noted in the posterior medial soft tissues of the distal tibia, which demonstrated intense enhancement and abuts the tibial nerve. This was measured approximately 2.2 cm in maximum craniocaudad dimension (Figure 4) and approximately 1.9 × 1.0 cm^2^ in maximum axial dimension (Figure 4). Preoperative biopsy was performed indicative of the mass to be schwannoma.

**Figure 5.**
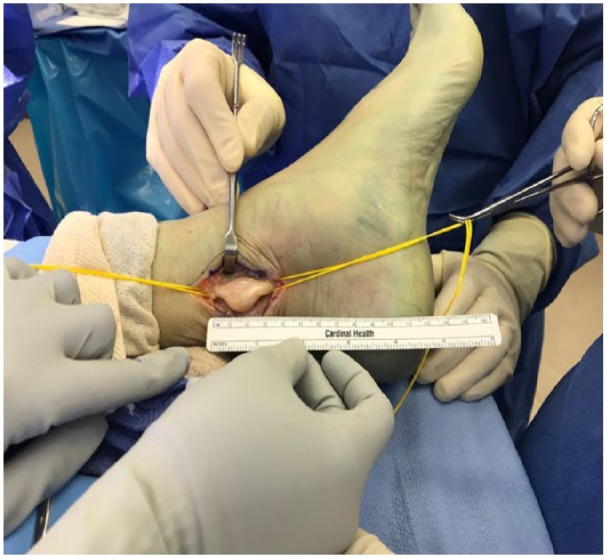
Surgical exposure and dissection of tibial nerve. The mass is encapsulated together with the nerve.

After careful assessment in a multidisciplinary team, the patient was referred to a specialist foot surgeon, and the tumor was surgically removed under general anesthesia. Prior to start of the surgical procedure an ultrasound was used to localize the area of the tibial nerve lesion, which was then marked using a skin marker. A longitudinal incision was made 1 cm wide behind the medial malleolus in line with the tibial nerve to expose the fascia of the tibial nerve. An enlarged mass of posterior nerve was seen. A freer elevator was used to lift the fascia away from the nerve and neurovascular bundle. Meticulous dissection was carried on to reserve the functional nerve fibers. A hemostat and blunt tenotomy scissors were used to dissect the nerve lesion, and the epineurium was gently incised longitudinally running in line with the nerve fibers and was dissected and retracted extracapsularly. The epineural tissue layer was meticulously dissected. It was found that the tumor surface can be exposed and the whole tumor can be freed via blunt dissection from the vascular bundle posterior medial distal leg (Figure 5).

**Figure 6.**
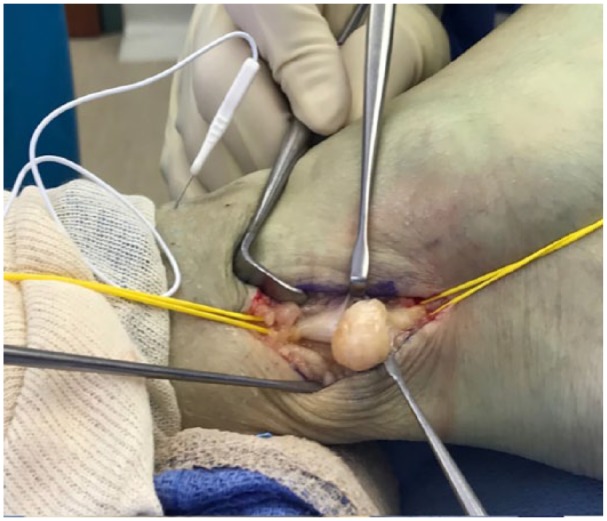
Tibial nerve schwannoma dissection from surrounding epineurium.

**Figure 7.**
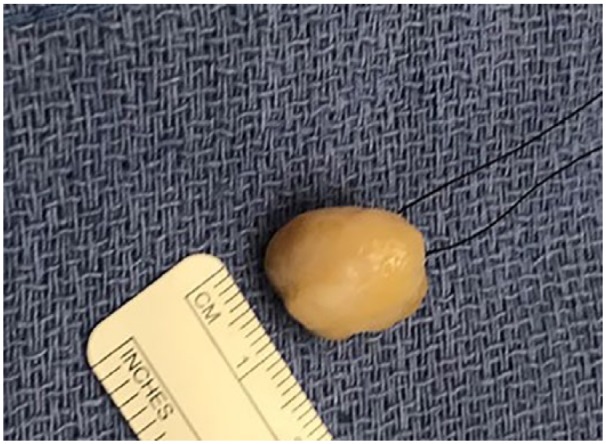
Schwannoma mass with size matching the measures in MRI.

Gentle dissection along the plane of the tumor capsule from the epineural layers allowed the tumor to be shelled out as a whole (Figure 6). The proximal and distal poles were approached, and two fascicles were isolated and cut from the nerve. A nerve stimulator (Vari-Stim III, nerve locator; Medtronic Xomed Inc, Jacksonville, FL, USA) was used to interrogate the nerve to identify the inactive nerve fascicles. A well-encapsulated tumor and inactive nerve was dissected and separated from the active nerve (Figure 7). The tumor was an approximately 22 × 19 × 10 mm^3^ well-encapsulated and circumscribed lesion. The rest of the posterior nerve was reserved. The specimen was sent for histopathological analysis. Next, a nerve stimulator was used again to check the motor function of the foot, which was noted to be intact.

**Figure 8.**
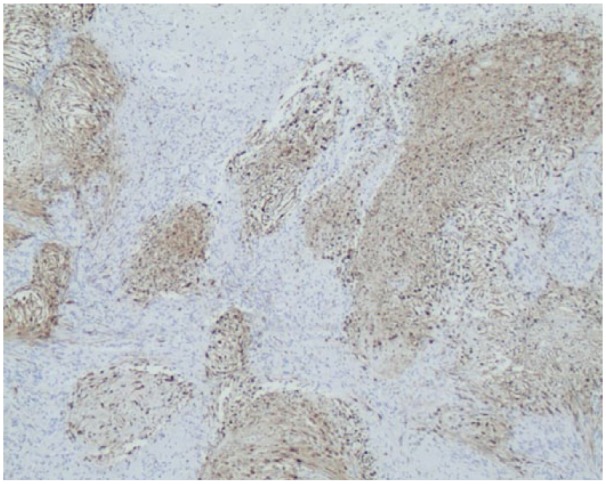
S100 positive immunostaining of schwannoma with diffused dark brown-stained tumor cells (magnification ×40).

**Figure 9.**
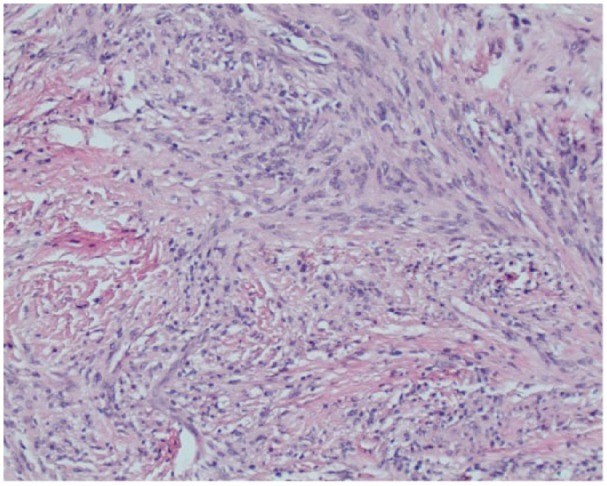
H&E stain of schwannoma (magnification ×100). Short fascicles of spindle cells with eosinophilic cytoplasm with syncytial appearance were found. These spindle cells were arranged in a vague palisade pattern. On immunohistochemistry, these spindle cells were immunoreactive to S100 protein.

The postoperative histopathological analysis of the specimen revealed bland spindle cells and hyalinized stroma. Majority of the spindle cells were strongly immunoreactive to S-100. A few cells were immunoreactive to epithelial membrane antigen (EMA) (Figures 8 and 9). Neurofilament (NF) immunostaining showed scattered entrapped axons. The findings were consistent with the diagnosis of peripheral nerve schwannoma.

At 12 months of follow-up after surgery, the patient was asymptomatic without pain or numbness and fully able to walk without any limitation. No instability or muscular weakness was present.

## Discussion

In the present case study we report a rare case of multiple myeloma accompanied with posterior tibial nerve schwannoma. Schwannomas can make up to 5.2% of all benign soft tissue tumors in all areas of the body, and its predilection to the foot and ankle region has a rate of 0.3% among all benign soft tissue tumors in the body.^4,5^ Peripheral schwannoma that coexisted with multiple myeloma has not been reported in the literature. Our case is the first case report regarding multiple myeloma accompanied with posterior tibial nerve schwannoma.

Our surgical treatment indicates that the posterior tibial nerve schwannoma can be encapsulated with the nerve trunk that appeared to be a whole piece of mass; however, meticulous dissection of the tumor separates the circumscribed lesions from the functional nerve trunk, and thus reserves other normal functional nerve fibers. A nerve stimulator can be used to interrogate the nerve to identify the inactive nerve fascicles and save active nerve fibers.

Schwannomas are nerve sheath nonmalignant tumors. Schwannomas may not need treatment if they are not causing any symptoms. Surgery is needed if the tumor is pressing on a nerve causing pain or other problems. In this case, the patient suffered significant pain; thus, surgery was performed and after surgery patient is free of pain now without neural deficit. Preoperative imaging and ultrasonic scanning are important for lesion localization but cannot differentiate between schwannoma and other lesions such as neurofibrosarcomas, which is a malignant tumor. Preoperative biopsy can be performed for the differential diagnosis.

A schwannoma typically comes from a single bundle (fascicle) within the main nerve and displaces the rest of the nerve. When a schwannoma grows larger, more fascicles are affected, making removal more difficult.^6^ In our case, surgical procedures demonstrated that this patient’s schwannoma is a well-encapsulated tumor that is adjacent to, but not associated with, the surrounding nerve fascicles; hence, surgical resection of the schwannoma mass does not damage majority of nerve fibers or lead to neural dysfunction. A nerve stimulator can be used intraoperatively to identify an area of inactive fascicles and avoid damaging a functional area of active fascicles when making the epineural incision, thus minimizing the sensory and motor function loss of the tibial nerve from the surgery.

Preoperative imaging and ultrasonic scanning are important for intraoperative localization. PET/CT scan can be used for tumor diagnosis and MRI can be used to delineate the anatomy of the tumor and localize it relative to the surrounding structures. A nerve stimulator was used intraoperatively to identify an area of inactive fascicles and avoid damaging a functional area of active fascicles when making the epineural incision, thus minimizing the sensory and motor function loss of the tibial nerve from the surgery.

It is not clear if there is an association between multiple myeloma and the development of schwannoma. On immunohistochemistry stains, the spindled cells displayed diffuse, strong nuclear and cytoplasmic positivity for S100 with a few cells positive with EMA staining indicative of locally formed myelin sheath neoplasm, hence ruling off the metastasis of multiple myeloma to the peripheral posterior tibial nerve. The relationship between multiple myeloma and peripheral nerve schwannoma is still inconclusive and remained to be elucidated.

Limitation of this study is that ultrasound scanning was not used for this patient. Ultrasound is widely used in the musculoskeletal systems. Ultrasound can be also used for scanning the nerve^7,8^ and might be more suitable than MRI for following up the case and cost-effective.

## Conclusion

We presented a case of posterior tibial nerve schwannoma with coexisting multiple myeloma under chemotherapy. The tibial nerve schwannoma appeared to be a posterior nerve mass, but meticulous dissection showed that this tibial nerve schwannoma was a well-encapsulated tumor. The tumor was resected with the rest of the nerve being reserved. Removal of the tumor made patient free of pain and asymptomatic after surgery.
